# Van der Waals Magnetic Tunnel Junctions Based on Two-Dimensional 1T-VSe_2_ and Rotationally Aligned h-BN Monolayer

**DOI:** 10.3390/nano15161246

**Published:** 2025-08-14

**Authors:** Qiaoxuan Zhang, Cong Wang, Wenjie Wang, Rong Sun, Rongjie Zheng, Qingchang Ji, Hongwei Yan, Zhengbo Wang, Xin He, Hongyan Wang, Chang Yang, Jinchen Yu, Lingjiang Zhang, Ming Lei, Zhongchang Wang

**Affiliations:** 1 Department of Electrical Engineering and Automation, Hebei University of Water Resources and Electric Engineering, Cangzhou 061001, China; zhangqiaoxuan@hbwe.edu.cn (Q.Z.); zhengrongjie@hbwe.edu.cn (R.Z.); jiqingchang@hbwe.edu.cn (Q.J.); yanhongwei@hbwe.edu.cn (H.Y.); wangzhengbo@hbwe.edu.cn (Z.W.); hexin@hbwe.edu.cn (X.H.); wanghongyan@hbwe.edu.cn (H.W.); yangchang@hbwe.edu.cn (C.Y.); yujinchen@hbwe.edu.cn (J.Y.); 2 Joint Training Program, Chongqing University of Posts and Telecommunications and Guidance Environmental Emergency Technical Equipment Research Center (Chongqing) Co., Ltd., Chongqing 400065, China; 3 College of Mathematics and Physics, Beijing University of Chemical Technology, Beijing 100029, China; wangcongphysics@mail.buct.edu.cn; 4 College of Science, China Agricultural University, Beijing 100083, China; wenjie.wang@cau.edu.cn; 5 International Iberian Nanotechnology Laboratory (INL), 4715-330 Braga, Portugal; rongsty@foxmail.com; 6 Multi-Agent Control Theory Center, Chongqing University of Posts and Telecommunications, Chongqing 400065, China; zhanglingjiang@cqupt.edu.cn; 7 School of Integrated Circuits, Beijing University of Posts and Telecommunications, Beijing 100876, China; 8 School of Materials and Energy, Southwest University, Chongqing 400715, China

**Keywords:** magnetic tunnel junctions, twist-angle engineering, van der Waals, 1T-VSe_2_

## Abstract

Magnetic tunnel junctions (MTJs) are pivotal for spintronic applications such as magneto resistive memory and sensors. Two-dimensional van der Waals heterostructures offer a promising platform for miniaturizing MTJs while enabling the twist-angle engineering of their properties. Here, we investigate the impact of twisting the insulating barrier layer on the performance of a van der Waals MTJ with the structure graphene/1T-VSe_2_/h-BN/1T-VSe_2_/graphene, where 1T-VSe_2_ serves as the ferromagnetic electrodes and the monolayer h-BN acts as the tunnel barrier. Using first-principles calculations based on density functional theory (DFT) combined with the non-equilibrium Green’s function (NEGF) formalism, we systematically calculate the spin-dependent transport properties for 18 distinct rotational alignments of the h-BN layer (0° to 172.4°). Our results reveal that the tunneling magnetoresistance (TMR) ratio exhibits dramatic, rotation-dependent variations, ranging from 2328% to 24,608%. The maximum TMR occurs near 52.4°. An analysis shows that the twist angle modifies the d-orbital electronic states of interfacial V atoms in the 1T-VSe_2_ layers and alters the spin polarization at the Fermi level, thereby governing the spin-dependent transmission through the barrier. This demonstrates that rotational manipulation of the h-BN layer provides an effective means to engineer the TMR and performance of van der Waals MTJs.

## 1. Introduction

Due to the functionality of magnetic tunnel junctions (MTJs) as spin-torque diodes [1], high-frequency oscillators [2], hard disk drive (HDD) read heads, and innovative spin-transfer torque magneto resistive random-access memory (STT-MRAM) devices [3], they have received substantial attention in the domain of spintronic devices. Alongside scientific advancements, new requirements for device integration have arisen, necessitating the development of novel materials and structures to miniaturize MTJ sizes while enhancing device performance. One approach is by constructing two-dimensional (2D) MTJs through van der Waals heterostructures stacking 2D ferromagnetic and non-ferromagnetic layered materials [4]. Despite their atomic thickness, these 2D ferromagnets maintain spin polarization in devices. The interlayer magnetic couplings in stacked ferromagnetic structures can be manipulated to control transport properties, enabling colossal magnetoresistance or TMR effects. Experimentally, magnetic materials that retain their magnetic properties after exfoliation from three-dimensional bulk materials include 1T-VSe_2_ [5], CrI_3_ [6], and Fe_3_GeTe_2_ [7]. Further, monolayer 1T-VSe_2_ magnetism occurs even at room temperature [5,8]. The discovery of these magnetic 2D materials has triggered extensive research on 2D MTJs and spin valve devices in spintronics.

The weak interlayer interactions in two-dimensional van der Waals heterostructures due to the vdW’s nature alleviate the experimental constraints of lattice matching between layers, thereby allowing various stable stacking configurations with diverse interlayer orientations. Experimentally accessible twist angles are more readily achievable in such stable rotation structures within the heterojunctions, where distinct rotation interfaces can be realized experimentally. These different interfaces exhibit unique transport characteristics. For example, twisted structures with angles of 0° and 60° have been synthesized in a two-dimensional system by growing monolayer MoS_2_ onto an h-BN surface using CVD [6], and graphene [9,10] has been rotationally aligned with an h-BN layer at 0° or 30° via thermally induced techniques [11]. Moreover, different helical fault types in WSe_2_ bilayers can be tuned experimentally by adjusting the precursor ratios (powders of selenium and tungsten oxide) in CVD reactions [12]. Twist-induced Moiré effects influence interfacial electromagnetic coupling, resulting in the modulation of electrical, optical, and magnetic properties of the heterostructures through rotational manipulation.

**Figure 1:**
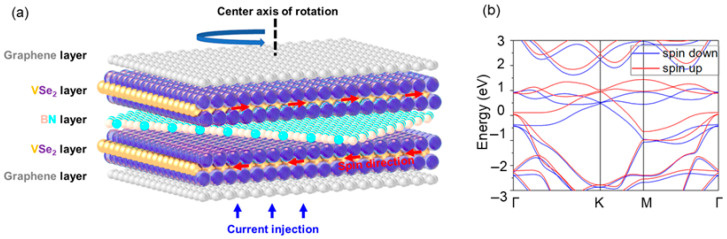
(**a**) The model structure of the van der Waals magnetic tunnel junction adopts a graphene/1T-VSe_2_/h-BN/1T-VSe_2_/graphene heterostack, with graphene serving as top and bottom electrode layers and where a monolayer h-BN insulating spacer is sandwiched between two ferromagnetic 1T-VSe_2_ monolayers. This vertically stacked heterostructure, facilitated by van der Waals bonding, supports the preferential vertical injection of carriers, as indicated by the blue arrow. Two configurations for the relative magnetic orientations of the two 1T-VSe_2_ layers are identified: a parallel configuration (PC) with aligned magnetizations and an anti-parallel configuration (APC) with opposing magnetizations. The APC direction is exemplified in the figure with a red arrow. (**b**) Band diagrams, accounting for spin effects, are computed using the generalized gradient approximation (GGA) in conjunction with the projector augmented wave (PBE) method for the monolayer VSe_2_ in its 1T phase.

Building upon the experimental advances outlined above, we employ DFT complemented by NEGF formalism to simulate and investigate the impact of rotation in an intermediate barrier layer on the performance and governing principles of a vertically stacked two-dimensional magnetically tunneling junction composed of graphene/1T-VSe_2_/h-BN/1T-VSe_2_/graphene, with a schematic representation schematically illustrated in Figure 1a. We systematically study how the transmission coefficients of the magnetic tunnel junction (MTJ) are influenced by rotating the h-BN layer, modeling 18 devices with interlayer angular intervals of approximately 10°. Our results reveal that the rotation of the h-BN layer leads to substantial variations in the tunneling magnetoresistance (TMR) ratio, ranging from 2328% to 24,608%. To probe the origin of this drastic change in TMR, we compute the projected density of states (PDOS) for graphene/h-BN/1T-VSe_2_ heterostructures with the h-BN rotated by 62.5° and 83.7° relative to the 1T-VSe_2_ heterojunction. Additionally, we obtain the spin-resolved local density of states’ (LDOS) distributions and K_∥_-space transmission coefficient maps for both spin-paralleled and spin-antiparalleled configurations of the MTJs. By doing so, we gain detailed insights into the behavior difference induced by the h-BN rotation and uncover the underlying influential factors.

## 2. Materials and Methods

Structural optimization of the graphene/1T-VSe_2_/h-BN heterostructures is performed using QuantumATK-2019.03 package [13,14], a density functional theory (DFT) [15]-based computational package. Electronic properties are calculated with the GGA-PBE functional [16,17] and a tuned on-site Hubbard parameter U = 1 eV for V 3d orbitals [18]. A plane-wave basis cutoff of 600 eV is employed alongside a Monkhorst-Pack k-point mesh (spacing: 0.01 Å^−1^) across the Brillouinzone (BZ). Forces are considered converged when reaching values less than 0.01 eV/Å^−1^.

Quantum transport simulations for MTJs utilize the QuantumATK engine, combining the nonequilibrium Green’s function (NEGF) method [19] with spin-polarized DFT under the spin-polarized generalized gradient approximation including an on-site Hubbard corrections (SGGA + U) approximation [20,21]. A double-zeta polarized (DZP) basis set [22] and real-space mesh cutoff of 100 Hartree are employed. Device calculations use periodic boundary conditions in transverse (x, y) directions at 300 K, with charge transport along the z-axis.

Optimized structures stabilize once convergence thresholds are met, with the maximum residual forces kept below 0.01 eV/Å^−1^ and energy convergence at 5 × 10^−6^ eV per atom. For the electrode and device central regions, k-point grids’ densities are 0.01 Å^−1^ along both a and b orientations, while the Monkhorst-Pack k-point grid for the transport direction is 100, corresponding to a range of h-BN rotation angles. Transmission coefficient $T_{\sigma }\left(E\right)$ is calculated in 2D first BZ, in a plane perpendicular to the transport directions. Mathematically, one can define $T_{\sigma }\left(E\right)$ as [23]:

(1) $$T_{\sigma }\left(E\right)\;=\;\frac{1}{\Omega_{BZ}}\;\int_{BZ}{dk_{//}T}_{\sigma }^{k_{//}}\left(E\right)$$

$\Omega_{BZ}$ means the volume of first 2D BZ, and $T_{\sigma }^{k_{//}}\left(E\right)$, representing the transmitted electrons through the MTJ, where $k_{//}$ designates in-plane reciprocal lattice vectors along the irreducible BZ boundary and *σ* denotes the spin state, can be analyzed in line with the approaches described in [24,25,26]. These coefficients help to unravel the transport behavior in various rotated h-BN heterostructures.

(2) $$T_{\sigma }^{k_{//}}\left(E\right)=Tr[\Gamma_{l,\;\sigma }^{k_{//}}(E){\;G}_{\sigma }^{k_{//}}\left(E\right){\;\Gamma }_{r,\;\sigma }^{k_{//}}\;\left(E\right){\;G}_{\sigma }^{k_{//}†}\left(E\right)\;]$$

The expressions $G_{\sigma }^{k_{//}}(E)$ and $G_{\sigma }^{k_{//}†}(E)$ denote the advanced and retarded Green’s functions, respectively. Given energy *E*, the quantity *T*(*E*) is averaged over all distinct $\sum_{l/r,\;\sigma }$ terms: $\Gamma_{l/r,\;\sigma }^{k_{//}}\left(E\right)\;=\;i(\sum_{l/r,\;\sigma }-\sum_{l/r,\;\sigma }^{†})$. The spin-resolved drain current *I_σ_* is computed using the Landauer–Büttiker formula [23,27,28]:

(3) $$I_{\sigma }\;=\;\frac{e}{h}\int_{-\infty }^{+\infty }\left\{T_{\sigma }\left(E\right)\left[{\;f}_{S}\left(E\;-\;\mu_{S}\right)\;-\;f_{D}\left(E\;-\;\mu_{D}\right)\right]\right\}dE$$

Here, *f_S_* and *f*_D_ denote the Fermi–Dirac distribution functions of the source and drain, respectively, while *μ_S_* and *μ*_D_ represent the Fermi levels of the source and drain electrodes. *T_σ_* signifies the transmission coefficient for spin component *σ*. All the transmission spectra computed in this work are based on the collinear spin configuration.

## 3. Results

### 3.1. Device Structure of Van der Waals Magnetic Tunnel Junction

Within the magnetic tunnel junction, graphene acts as both the left and right metallic electrode layers, while 1T-VSe_2_ serves as the ferromagnetic layer. By rotating the h-BN layer, we investigate the influence of barrier rotation on device performance. The 1T phase of VSe_2_ is a magnetic transition metal dichalcogenide [5]; the band structure for a single-layer, displayed in Figure 1b, corresponds with previous research [29]. Its lattice parameter is a = b = 3.44 Å [2], while the h-BN barrier has a lattice constant of a = b = 2.51 Å. Owing to the ability to grow monolayer 1T-VSe_2_ on graphene or MoS_2_ substrates via molecular beam epitaxy, we adopt single-layer graphene as the metal electrode layer adjacent to the VSe_2_ layer. To avoid additional strain complications, only graphene’s lattice parameter is stretched to match that of VSe_2_. The persistence of electronic states of graphene layers in the projected density of states’ diagrams of devices rotated 62.5° and 83.7° in Figures 5 and 6, close to the Fermi level, verifies that strained graphene still retains its metallic nature. In our work, we rotationally manipulate the h-BN layer while maintaining the same stacking configuration between VSe_2_ and the strained graphene layers across all devices to minimize varying parameters. No pinning layers are included in our MTJs; all devices are configured with a basic MTJ structure.

We fabricate 18 magnetic tunnel junction devices, controlling the h-BN layer’s rotation relative to the 1T-VSe_2_ layer over a range of approximately 0° to 180° in increments of 10°, ensuring a lattice mismatch of less than 3% for all heterostructures. The lattice parameters for all constructed structures are displayed in Table 1. For comparison purposes, we hold the 1T-VSe_2_ and graphene layers at the same angles and positions while manipulating the orientation of the h-BN layer. Specifically, we highlight our rotation model with two examples, viz., graphene/1T-VSe_2_/h-BN structures rotated at 62.5° and 83.7°.

**Figure 2:**
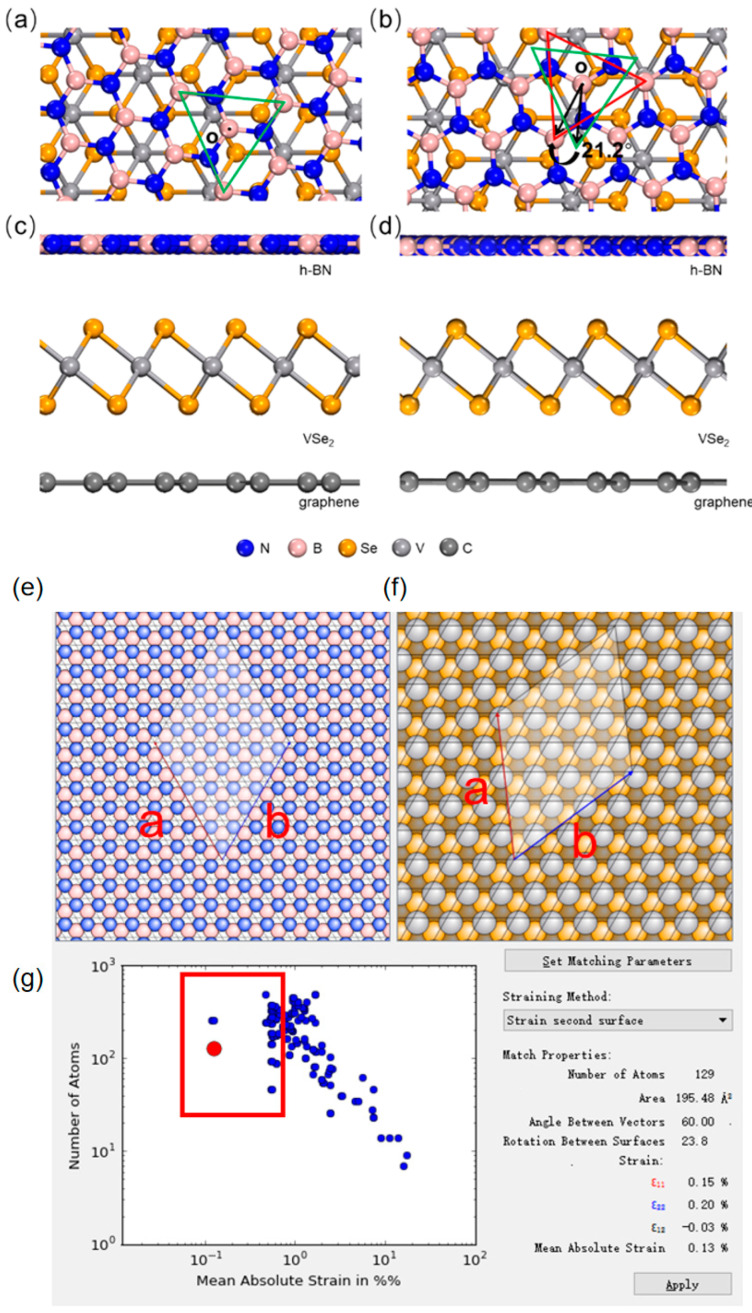
The models of heterojunctions within an h-BN/1T-VSe_2_/graphene heterostructure. (**a**) The top view of a model depicting the h-BN layer rotated 62.5 degrees relative to the 1T-VSe_2_ layer. (**b**) A top view of another model showing the h-BN layer rotated at an angle of 83.7 degrees compared to the 1T-VSe_2_ layer. Green and red triangles represent the distribution angles of Boro atoms in (**a**,**b**), respectively. The orientation of the VSe_2_ layer remains identical in both (**a**,**b**). (**c**,**d**) These present the side views of the heterojunctions corresponding to the 62.5-degree and 83.7-degree rotation models, respectively. In model (**a**), a green triangle is used to highlight the 62.5-degree angle of the h-BN layer, which aligns with the green triangle in the same position. In model (**b**), a red triangle marks the identical boron atom structure found in the h-BN layer at the position of the aforementioned green triangle. The centers of the two triangles (labeled as point o) coincide, clearly indicating that the h-BN layer in the 83.7-degree model is rotated clockwise by 21.2 degrees relative to the structure depicted in the 62.5-degree model. (**e**,**f**) These depict the monolayer structures of h-BN and VSe_2_, respectively, illustrating the 23.8° rotational configuration arbitrarily selected for subsequent heterostructure modeling. Both monolayers exhibit matched lattice constants a and b at this orientation. The lattice parameters *a* and *b* define the unit cell dimensions along orthogonal crystallographic axes. (**g**) Left panel displays all possible rotational configurations exhibiting lattice mismatch. Each point in the figure represents an interfacial composite configuration. Only configurations with mean absolute lattice mismatch < 3% (right panel) are selected to ensure accuracy, restricting viable models to the red-outlined region on the (**g**) left.

In the top–down view of the 62.5° heterojunction depicted in Figure 2a, equilateral green triangles identify a section of h-BN atoms. Equivalent h-BN atoms are framed by red equilateral triangles in the top–down view of the 83.7° stacking schematically illustrated in Figure 2b. To facilitate comparison, we duplicate the same-angle green triangle from (a) onto (b) and align their centroids O, illustrating that in the 83.7° heterojunction, the h-BN layer is rotated counterclockwise by 21.2° relative to that in the 62.5° structure. Additional evidence is provided by the side views in Figure 2c,d, revealing no relative rotation between the 1T-VSe_2_ and graphene layers in either heterojunction, with the rotation confined solely to the h-BN layer.

Employing QuantumATK software and DFT, we optimize the stable interlayer spacings within the devices. Interlayer distances: 3.53 Å (graphene-graphene), 3.22 Å (graphene-VSe_2_), and 3.48–3.54 Å (VSe_2_-h-BN, except 3.73 Å at 10.8°). These specific spacings can be found in Table 1.

### 3.2. Electrical Properties of Rotating h-BN Layer in the Magnetic Van der Waals Tunnel Junctions

We construct a graphene/1T-VSe_2_/h-BN/1T-VSe_2_/graphene MTJ configuration and subject it to an external magnetic field to manipulate the magnetic moments of the two ferromagnetic layers into either parallel spin alignment or antiparallel alignment. In the parallel spin configuration, the tunneling resistance significantly drops compared to the resistance when they are in the antiparallel state. The normalized difference between these two resistances defines the tunnel magnetoresistance ratio (TMR). Assuming spin conservation during tunneling, we employ the most optimal definition to describe TMR:

(4) $$\mathrm{TMR}=\frac{G_{\mathrm{PC}}-G_{\mathrm{APC}}}{G_{\mathrm{APC}}}\times 100\%$$

The conductivity can be calculated using the following formula:

(5) $$G_{\mathrm{PC}}=\frac{e^{2}}{h}\left(T_{↑}^{\mathrm{PC}}+T_{↓}^{\mathrm{PC}}\right)$$

(6) $$G_{\mathrm{APC}}=\frac{e^{2}}{h}\left(T_{↑}^{\mathrm{APC}}+T_{↓}^{\mathrm{APC}}\right)$$

*G*_PC_ refers to the conductance of the MTJ with parallel spin orientation, while *G*_APC_ represents the conductance with antiparallel alignment. $T_{↑}^{PC}$($T_{↑}^{APC}$) and $T_{↓}^{PC}$($T_{↓}^{APC}$) denote the parallel (antiparallel) transmission coefficients for spin-up and spin-down electrons, respectively. Similarly, the TMR effect can also be described in terms of the device’s resistance according to Equation (3), which expresses the TMR ratio as follows:

(7) $$\mathrm{TMR}=\frac{R_{\mathrm{PC}}-R_{\mathrm{APC}}}{R_{\mathrm{APC}}}$$

**Figure 3:**
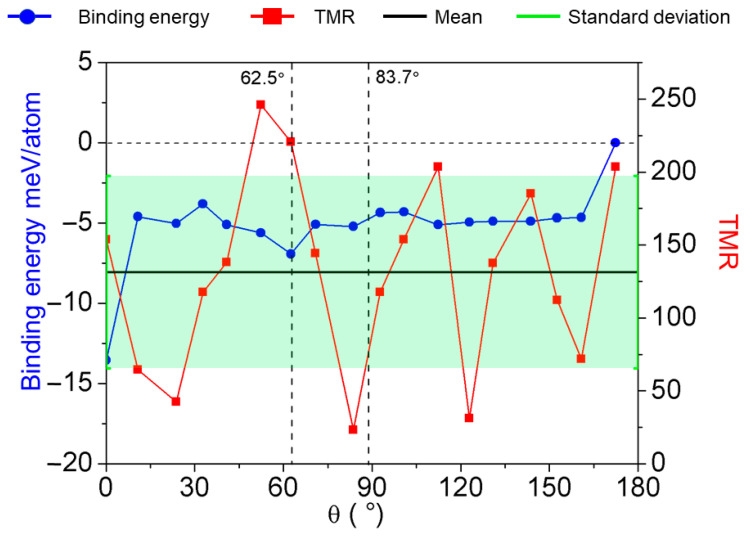
The distribution of binding energies at the 1T-VSe_2_/h-BN interface is depicted by the blue line, corresponding to the h-BN layer rotated at various angles. The red dotted line shows the distribution of TMR for MTJs rotated at different angles. The vertical axis for the binding energy represented by the blue line is on the left side, whereas the vertical axis for the TMR represented by the red line is on the right side. The solid black line indicates the mean value (TMR = 131.54), while the green vertical bars represent the standard deviation (SD = 66.02). The range of SD variation is delineated by the light green shaded area.

*R*_PC_ and *R*_APC_ represent the resistance in the spin-parallel and spin-antiparallel states, respectively. Based on the mentioned formula, to calculate the rotation effect of h-BN layers on the tunneling resistance and conductance of two-dimensional MTJs, we perform calculations of device transmission spectra at zero bias for MTJs with various h-BN layer rotational angles. A summary of the parallel and antiparallel conductance values, along with their respective resistances and TMR, is compiled in Table 1 for devices with different rotation angles. The red curve in Figure 3 illustrates the variation of TMR with h-BN layer rotation and demonstrates a wide range from 2328% to 24,680%. There is no evident pattern between the h-BN angular rotation within the devices and the distribution of TMR values. The device with the highest TMR performance corresponds to the one with a 52.4° rotation angle. The blue curve in Figure 3 presents the binding energy of each atom within the 1T-VSe_2_/h-BN heterostructure for differently rotated devices. The formation energy *E*_form_ within the interface of the heterojunction can be expressed as follows:

*E*_form_ = *E*(VSe_2_/h-BN)-*E*(VSe_2_)-*E*(h-BN) (8)

*E* (VSe_2_/h-BN) represents the total energy after stacking of the 1T-VSe_2_/h-BN structure, while *E* (VSe_2_) and *E* (h-BN) denote the energies of a single-layer 1T-VSe_2_ and h-BN, respectively, in a vacuum environment. A higher binding energy signifies increased instability of the rotational configuration. We set the 1T-VSe_2_/h-BN binding energy of the most stable structure, at 172.4°, to 0 meV/atom, against which we gauge the binding energies of other h-BN rotation structures.

Our calculated interface binding energies for the various rotation angles of the 1T-VSe_2_/h-BN heterostructures shown in Figure 3 mainly cluster between −3.8 meV/atom and −6.9 meV/atom, suggesting that an appreciable bonding interaction exists between 1T-VSe_2_ and h-BN layers across these angles. Specifically, the 0° structure has the lowest interface binding energy of −13.5 meV/atom. While overcoming an interface binding energy difference of 8.9 meV/atom is required for h-BN to rotate from 0° to 10.8°, and a shift from 160.8° to 172.4° involves overcoming 4.7 meV/atom, the clockwise rotation of h-BN from 10.8° to 160.8° is relatively facile. Considering the experimentally observed 52.4° structure with the highest TMR is prone to slide to the 62.5° structure with lower local interface binding energy, indicated by a black dashed line in Figure 3, we highlight the interface binding energy and TMR characteristics of these two devices (62.5° with the second-highest TMR and 83.7° with the lowest TMR) for more profound investigations into the interplay between electric and magnetic properties due to h-BN layer rotations.

**Figure 4:**
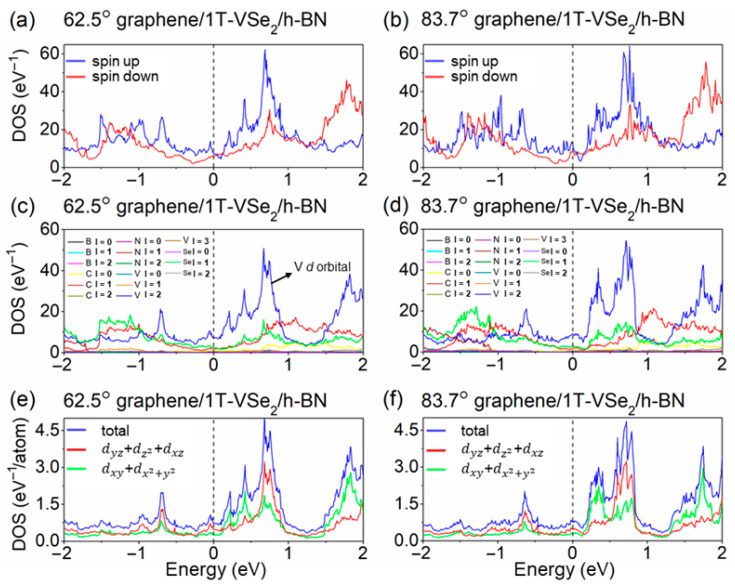
(**a**,**b**) These depict the spin-up and spin-down density of states for graphene/1T-VSe_2_/h-BN heterostructures at rotation angles of 62.5° and 83.7°, respectively, when spin effects are considered; panels (**c**,**d**) illustrate the projection of orbital angular momentum quantum numbers (l = 0; 1, 2, 3 corresponding to s, p, d, and f orbitals, respectively) for the density of states in these heterostructures at the same rotation angles. Panels (**e**,**f**) focus specifically on the projection of the density of states for the d-orbital angular momentum of vanadium (V) atoms within the 1T-VSe_2_ layer at the aforementioned rotation angles. Green lines outline the density of states for the $d_{xy}$ + $d_{x^{2}{+y}^{2}}$ orbital within the plane of 1T-VSe_2_. Red lines depict the density of states for the orbital $d_{yz}$ + $d_{z^{2}}$ + $d_{xz}$ perpendicular to the plane of 1T-VSe_2_. The sum of these two densities of states, colored in blue, is also shown to provide a comprehensive view of the electronic behavior across these systems.

We proceed in Figure 4a,b to investigate the PDOS for the spin-up and spin-down channels of graphene/1T-VSe_2/_h-BN heterostructures at 62.5° and 83.7° after the rotation of h-BN. A distinct contrast in the electronic states near the Fermi level and other peaks in PDOS profiles for both heterostructures’ spin-up and spin-down channels is observed. It is well-known that magnetic properties in the heterostructure are predominantly due to the presence of unpaired electrons in V atoms’ *d* orbitals. The differences in PDOS peak distributions between the spin-up and spin-down channels suggest that rotation of the h-BN layer influences the magnetization direction within the 1T-VSe_2_ layer, thereby affecting electron states for the two spins. Moreover, the imbalance of spin-up and spin-down channel DOS in each heterostructure further indicates a spin-polarization phenomenon within the 1T-VSe_2_ layer that depends on the overall system.

Moving forward, in Figure 4c,d, we analyze the distribution of angular momentum quantum number l for distinct elements’ orbitals in the two rotated heterostructures. The third atomic d orbitals of the vanadium (V) atom in the 1T-VSe_2_ layer contribute significantly to the PDOS compared to other elements or other V orbitals at the Fermi level. Hence, we depict the projected DOS for individual V atom d orbitals of the 62.5° and 83.7° heterostructures, as schematically illustrated in Figure 4e,f. Notably, both V atoms exhibit similar d-orbital DOS distributions, with two groups: the out-of-plane $d_{yz}$ + $d_{z^{2}}$ + $d_{xz}$ orbitals and in-plane $d_{xy}$ + $d_{x^{2}{+y}^{2}}$ orbitals [3]. Analyzing the Fermi-level changes in V atom d-orbital states for the two angles confirms the DOS values for out-of-plane and in-plane d orbitals for the 62.5° structure are 0.47 eV^−1^/atom and 0.25 eV^−1^/atom, whereas for the 83.7° structure, they are 0.46 eV^−1^/atom and 0.29 eV^−1^/atom. Despite modest differences in these plane states at the Fermi level, there is a noticeable peak difference (62.5°: 0.72 eV^−1^/atom vs. 83.7°: 0.38 eV^−1^/atom at E = −0.04 eV) in out-of-plane DOS just above the Fermi level with much less variation in the in-plane orientation (within 0.09 eV^−1^/atom). These contrasting features may account for the influence on TMR for the two devices with distinct rotations.

**Figure 5:**
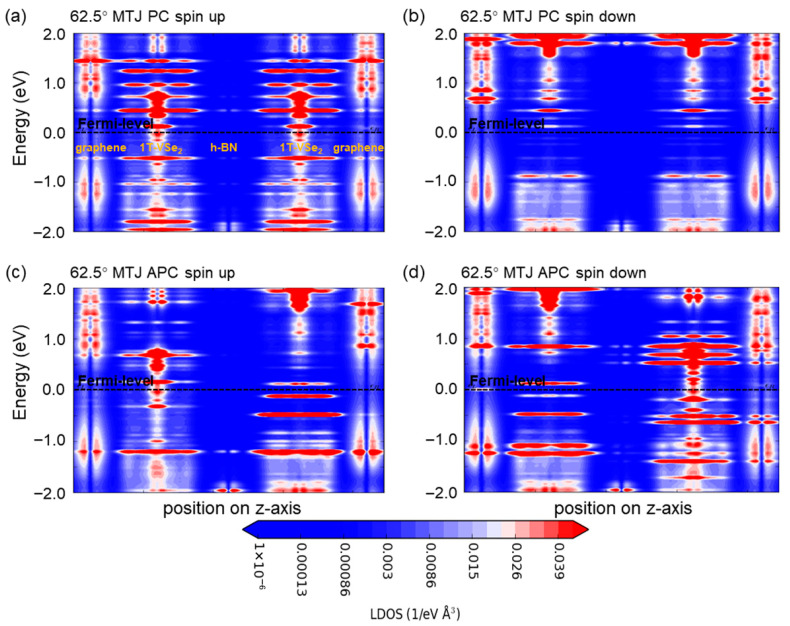
The local density of states’ (LDOS) maps are illustrated for van der Waals magnetic tunnel junction devices under hexagonal boron nitride (h-BN) rotation. (**a**) This shows the spin-up channel configuration for an h-BN rotation of 62.5° in a parallel spin geometry. (**b**) This depicts the spin-down channel configuration under the same 62.5° h-BN rotation, also in a parallel spin geometry. (**c**) This presents the spin-up channel configuration but in an antiparallel spin geometry with the 62.5° h-BN rotation. (**d**) This represents the spin-down channel configuration within the antiparallel spin geometry at 62.5° h-BN rotation.

**Figure 6:**
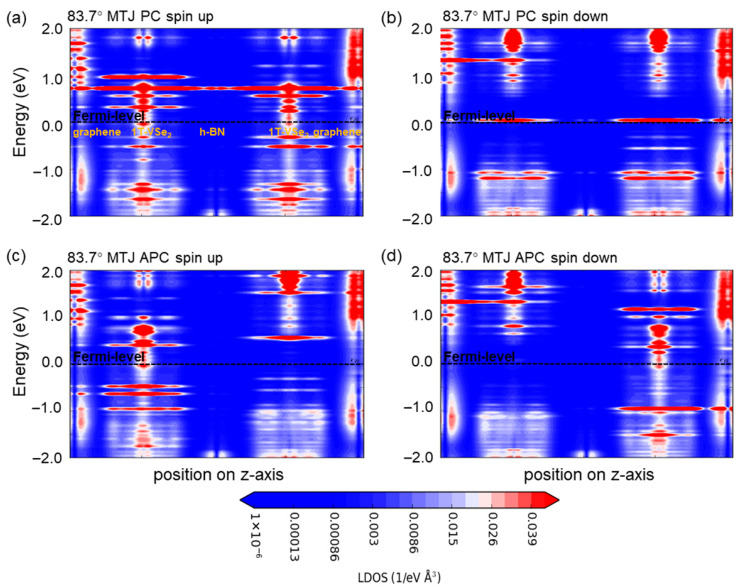
The local density of states’ (LDOS) maps for van der Waals magnetic tunnel junction devices under hexagonal boron nitride (h-BN) rotation are presented. (**a**) This displays the spin-up channel configuration for an h-BN rotation of 83.7° in a parallel spin geometry. (**b**) This illustrates the spin-down channel configuration under the same 83.7° h-BN rotation, also in a parallel spin geometry. (**c**) This showcases the spin-up channel configuration but in an antiparallel spin geometry with the 83.7° h-BN rotation. (**d**) This represents the spin-down channel configuration within the antiparallel spin geometry at 83.7° h-BN rotation.

Further, we utilize spin-resolved local density of states’ (LDOS) maps for the 62.5° (Figure 5) and 83.7° (Figure 6) devices to scrutinize the change in energy bands within the devices. Focusing on the central region’s LDOS images (neglecting those from the graphene electrodes) and highlighting the location of different material layers in Figure 5a and Figure 6a, it is confirmed that graphene’s gap at the Fermi level remains at 0 eV, preserving its metallic nature, while the h-BN layers retain insulating characteristics. Rotation of the h-BN layer alters the Fermi-level spin-up and spin-down channels of 1T-VSe_2_ and influences interface state distribution between the two, leading to dissimilar behavior between the devices.

**Figure 7:**
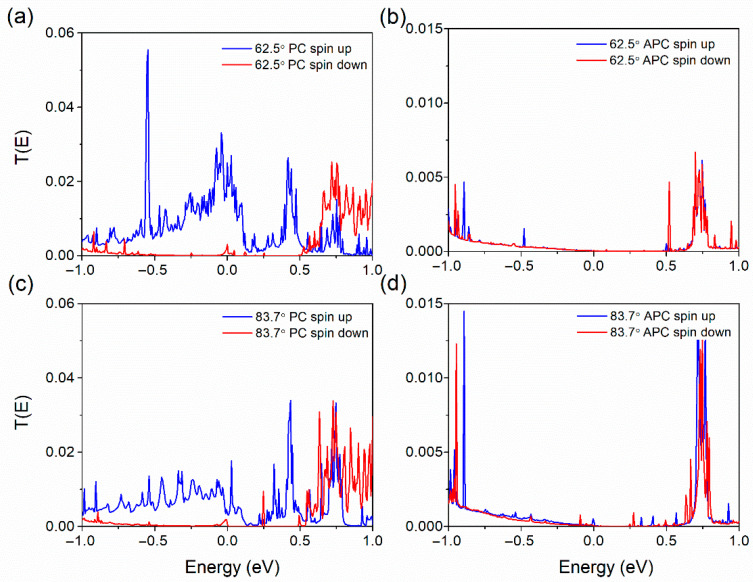
The transmission spectra of van der Waals magnetic tunnel junctions under hexagonal boron nitride (h-BN) rotation are delineated: (**a**) depicts the spectral characteristics for a spin-parallel configuration with a 62.5° h-BN rotation; (**b**) shows the spectral behavior for a spin-antiparallel configuration at the same 62.5° h-BN rotation angle; (**c**) features the transmission spectra for a spin-parallel configuration with an 83.7° h-BN rotation; (**d**) illustrates the transmission properties for a spin-antiparallel configuration under the 83.7° h-BN rotation angle.

Figure 7a,b present spin-resolved transmission spectra for MTJ with spin-parallel and antiparallel configurations in h-BN devices with a rotation angle of 62.5°. Similarly, Figure 7c,d illustrate the spin-resolved transmission spectra for those with a rotation angle of 83.7° in both parallel and antiparallel arrangements. In the parallel configuration, a pronounced contrast in transmission coefficients near the Fermi level is observed between the upward spin channel for 62.5° devices (0.025) and 83.7° devices (approximately 0.0033). Downward-spin channel transmissions for these angles exhibit comparably low values, specifically 0.003 for the 62.5° devices and 4.21 × 10^−4^ for the 83.7° devices. Under antiparallel configurations, both angle-rotated devices exhibit negligible transmission coefficients for both upward and downward spins, ranging from 3.87 × 10^−5^ to 4.21 × 10^−4^. Calculating spin polarization using the formula η = (*T*_↑_(E_f_) − *T*_↓_(E_f_))/(*T*_↑_(E_f_) + *T*_↓_(E_f_)), where *T*_↑_(E_f_) and *T*_↓_(E_f_) represent the transmission coefficients for the spin-up and spin-down channels at the Fermi energy level, yields a spin polarization rate of 78.18% for the 62.5° device’s parallel configuration and 77.66% for the 83.7° device’s counterpart. These results indicate that the 62.5°-rotated device facilitates effective spin injection in its parallel configuration, while spin injection of the Bloch states in the 83.7° device is relatively more challenging than in the 62.5° device. Conclusively, it can be inferred that the rotation of h-BN layers influences the device’s transmission characteristics, with variations also evident through the change in TMR values.

**Figure 8:**
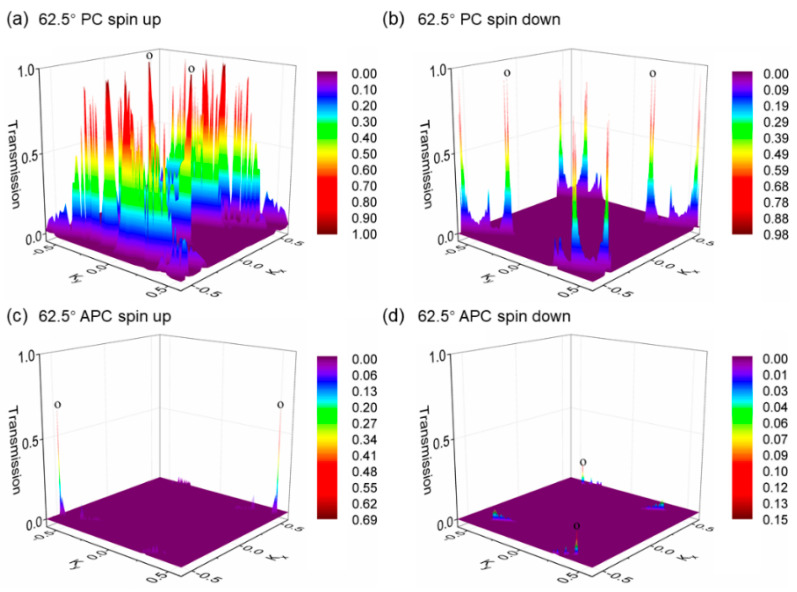
The two-dimensional Brillouin zone-resolved band structures for the van der Waals magnetic tunnel junctions with a 62.5° h-BN rotation are delineated, differentiated by the following: (**a**) the spin-up channel for the parallel spin configuration, (**b**) the spin-down channel for the parallel spin configuration, (**c**) the spin-up channel for the antiparallel spin configuration, and (**d**) the spin-down channel for the antiparallel spin configuration. The eigenvalue distribution maps display the distribution patterns for each channel. The hotspots, which denote the peaks of the transmission eigenvalues within these maps, are indicated using the letter ‘o’ in the figures. These points highlight areas of significant conductance changes and spin-specific interactions within the junctions as the orientation of the h-BN layer is adjusted through rotation.

Next, for a more meticulous understanding of the magnetic tunnel junction’s Fermi-level transmission spectrum, we present Figure 8, showing the k-resolved and spin-resolved transmission eigenvalue distribution in the vicinity of the Fermi surface for the 62.5° device. In the parallel spin configuration for the 62.5° rotation device, the spin-up channel exhibits high transmission eigenvalues in regions distant from the center of the two-dimensional BZ, indicating prominent interface states. Due to the larger distance from the Γ point, there is a higher decay coefficient, suggesting that increasing the number of h-BN layers would be disadvantageous to transmission. Conversely, for the spin-down channel, the interface state eigenvalues are significantly lower. In the antiparallel spin configuration, the eigenvalue distributions for the spin-up and spin-down channels are comparable, predominantly located around and at the Γ point; however, their eigenvalues are substantially weaker compared to the hotspots (points o) of the spin-up channel in the parallel configuration.

**Figure 9:**
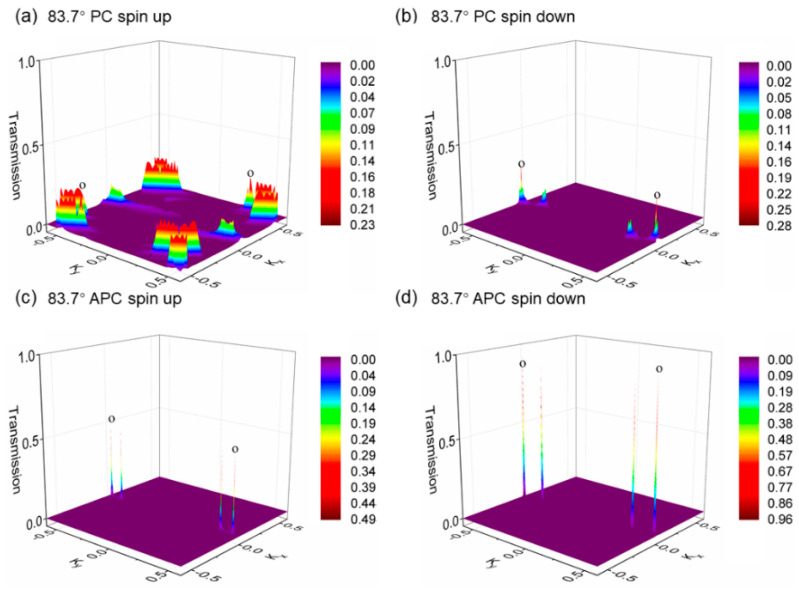
The two-dimensional BZ-resolved band structures for the van der Waals magnetic tunnel junctions with an 83.7° h-BN rotation are illustrated, characterized by the following: (**a**) the spin-up channel for the parallel spin configuration, (**b**) the spin-down channel for the parallel spin configuration, (**c**) the spin-up channel for the antiparallel spin configuration, and (**d**) the spin-down channel for the antiparallel spin configuration. The eigenvalue distribution maps depict the distribution patterns for each channel. The hotspots, which signify the peaks of the transmission eigenvalues within these maps, are marked using the symbol ‘o’ in the figures. These points underscore areas of notable conductance variations and spin-specific interactions within the junctions as the orientation of the h-BN layer is altered through rotation.

In Figure 9, we demonstrate the spin-resolved and k-resolved transmission eigenvalue distributions near the Fermi level in the two-dimensional BZ for the 83.7°-rotated h-BN device for both parallel and antiparallel alignments. Figure 9a reveals that the spin-up channel of the parallel spin configuration displays interface states significantly lower than those in the parallel spin-up channel of the 62.5° device, at an even greater distance from the Γ point, implying a higher attenuation factor. Furthermore, the interface states for the 83.7° device in both parallel spin-up and antiparallel configurations appear particularly sparse.

**Figure 10:**
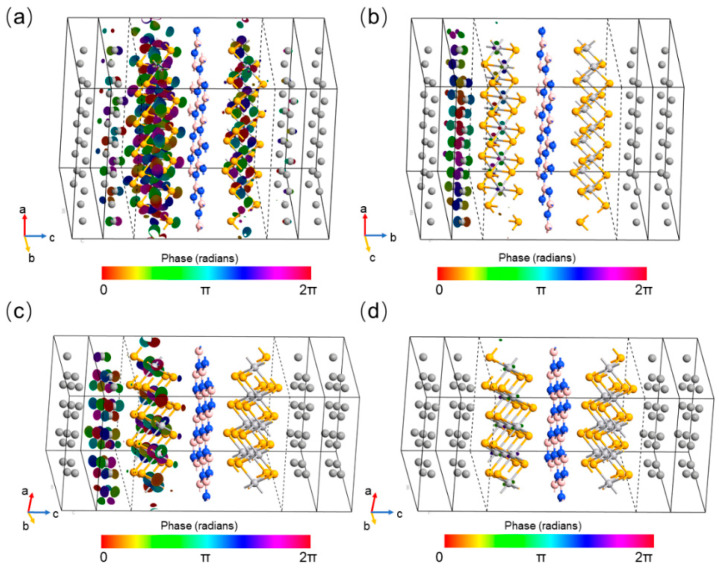
Figure 10 presents the spatial distribution of the eigenfunctions corresponding to the transmission spectra at the hotspots for the van der Waals magnetic tunnel junctions (MTJs) with a hexagonal boron nitride (h-BN) layer rotated to the following: (**a**) the spin-up channel and (**b**) the spin-down channel for an MTJ with a 62.5° rotation angle of h-BN; (**c**) the spin-up channel and (**d**) the spin-down channel for an MTJ with an 83.7° rotation angle of h-BN, within the devices. These eigenfunctions highlight the variation in transmission properties and spin-dependent interactions at the critical locations (hotspots) within the MTJs as influenced by the rotational orientation of the h-BN layer.

We proceed by plotting the transmission spectrum eigenfunctions’ distribution at the hotspots o in the two-dimensional BZ for the 62.5° device for the parallel spin-up channel (a) and the spin-down channel (b), and for the 83.7° device for the parallel spin-up channel (c) and the spin-down channel (d), as depicted in Figure 10. Clearly, as seen in Figure 10a, the Bloch states of the parallel spin-up channel in the 62.5° rotation device can penetrate the h-BN barrier to reach the 1T-VSe_2_ layer on the right. On the other hand, it is evident in Figure 10b that the Bloch states of the parallel spin-up channel in the 83.7° device have great difficulty in traversing the h-BN barrier. As schematically illustrated in Figure 10c,d, for the parallel spin-down channels in both configurations, the eigenfunctions barely extend to the 1T-VSe_2_ layer situated on the opposite side of the h-BN blocking layer. Regarding the antiparallel configurations with their lower eigenvalues, the Bloch states in the devices experience even greater difficulty in crossing the h-BN barrier, leading to comparably low Fermi surface transmission rates. The contrasting distribution of the transmission eigenstates in the parallel spin-up channels between these two devices implies that the 62.5° device has a smaller attenuation coefficient and hence permits easier passage of the Bloch states through the h-BN layer, which results in the significant difference in TMR values owing to the distinct magnetic resistances in each device.

Considering the outcome of our computational process, it becomes apparent that the rotation of the h-BN layer leads to dramatic changes in TMR. We select the 62.5° and 83.7° devices, with comparable stable h-BN/1T-VSe_2_ binding energies and significant TMR disparities, for a detailed analysis. First, we observe that the electronic state density of the valence d orbitals of V atoms in all the elements’ orbitals within the graphene/1T-VSe_2_/h-BN heterostructure carries the highest weight. This is because the presence of unpaired electrons in the *d*-orbital layer endows the 1T-VSe_2_ monolayer with its magnetic property. As the h-BN layer rotates, a distinct peak emerges in the planar-state density of the $d_{yz}$ + $d_{z^{2}}$ + $d_{xz}$ orbitals at an energy of −0.04 eV for the 62.5° heterojunction compared to that of the 83.7° junction, potentially impacting transmission at the Fermi level for the two devices. Furthermore, the local density of states (LDOS) for the spin-up and spin-down channels at the two different rotation angles of 1T-VSe_2_ show distinct characteristics at the Fermi energy level.

## 4. Discussion

Combining these observations, it is clear that the rotation of the h-BN layer indeed affects the electron distribution within the 1T-VSe_2_ layer, particularly influencing the electronic configuration of the outer d orbitals of the V atoms, hence altering the magnetization direction and magnetic resistance. Consequently, it comes as no surprise that the transmission coefficient for the spin-up channel in the 62.5° parallel configuration significantly exceeds that of the 83.7° configuration. To thoroughly investigate the underlying reasons behind the substantial TMR variation induced by the rotation of the h-BN layer, we have compared the behaviors of these two devices representing differing angles.

## 5. Conclusions

To investigate the impact of the twist angle in the h-BN blocking layers on device performance, we have constructed a device model comprising 18 graphene/1T-VSe_2_/h-BN/1T-VSe_2_/graphene MTJ, with the h-BN layer separated by roughly 10° rotations. The transmission spectra analysis of magnetic tunnel junctions (MTJs) with h-BN twisted at 62.5° and 83.7° reveals spin-polarization rates of 78.18% and 77.66%, respectively, for parallel spin configurations. Despite this minimal difference in polarization (<0.52%), the tunneling magnetoresistance (TMR) across a 0–172.4° twist range exhibits extraordinary variation—spanning 2328% to 24,608%. Our study reveals that rotation of the h-BN layer notably modifies the V atom’s *d*-orbital electronic state distribution within the 1T-VSe_2_ layer, leading to substantial differences in TMR. The high TMR values suggest that 1T-VSe_2_ combined with h-BN forms an excellent magnetic tunnel junction device, while the pronounced variation of TMR implies significant performance changes due to the twisted h-BN layers. Hence, guided by our findings, we demonstrate the tunability of magnetic tunnel junction device characteristics through rotation of the h-BN layer. We propose an approach for engineering different TMR magnitudes by manipulating the rotation angle of the blocking layer in van der Waals MTJ, offering guidelines for experiments.

## Figures and Tables

**Table 1:** The transmission spectra of the Fermi surface conductance for graphene/1T-VSe_2_/h-BN/1T-VSe_2_/graphene magnetic tunnel junctions with parallel and antiparallel configurations, under different angles of rotation *θ* for the h-BN layer, are presented. The table presents the conductance values in Siemens. The crystal lattice parameters a and b for both the a and b directions, the stable distance d between the h-BN layer and the 1T-VSe_2_ layer, and the TMR value (tunneling magneto-resistance ratio) in the final column, are given for each device heterostructure with its specific rotation angle.

*θ* (°)	*a* (Å)	*b* (Å)	*Γ* (°)	*G*_PC_ × 1 × 10^−7^ (Siemens)	*G*_APC_ × 1 × 10^−9^ (Siemens)	*d* (Å)	TMR%
0	6.65	6.62	60.04	1.64	1.06	3.53	15,372
10.8	8.70	8.70	119.99	0.78	1.19	3.73	6455
23.8	15.18	8.85	50.03	3.45	7.91	3.52	4262
32.8	8.90	8.89	22.32	3.80	3.20	3.54	11,775
40.8	6.62	6.66	60.30	1.46	1.05	3.54	13,805
52.4	16.47	8.83	39.4	7.19	2.91	3.48	24,608
62.5	28.47	11.72	16.30	9.60	4.33	3.50	22,071
70.9	8.72	8.66	59.91	3.88	2.67	3.53	14,432
83.7	15.18	8.85	50.03	2.20	9.06	3.49	2328
92.8	8.88	8.89	60.12	3.93	3.31	3.54	11,773
100.8	6.61	6.66	60.30	1.44	0.93	3.54	15,384
112.4	16.47	8.84	39.41	3.66	1.79	3.53	20,347
122.9	11.72	8.98	64.37	1.77	5.51	3.53	3108
130.9	8.72	8.66	59.90	3.06	2.21	3.53	13,746
143.7	15.18	8.89	129.73	6.11	3.28	3.53	18,528
152.6	15.42	8.88	89.91	7.49	6.61	3.54	11,231
160.8	6.62	6.63	60.02	0.99	1.36	3.53	7179
172.4	16.47	8.88	140.22	6.18	3.02	3.53	20,364

## Data Availability

The original contributions presented in this study are included in the article. Further inquiries can be directed to the corresponding authors.
